# Symptomatic and asymptomatic malaria prevalence and its determinant factors in pastoral communities of Waghemira Zone, Northeast Ethiopia: A community‐based cross‐sectional study

**DOI:** 10.1002/hsr2.1336

**Published:** 2023-06-07

**Authors:** Habtu Debash, Gebru Tesfaw, Hussen Ebrahim, Agumas Shibabaw, Yimer Melese, Mihret Tilahun, Ermiyas Alemayehu, Ousman Mohammed, Melkam Tesfaye, Mengistu Abate

**Affiliations:** ^1^ Department of Medical Laboratory Science, College of Medicine and Health Sciences Wollo University Dessie Ethiopia; ^2^ Department of Internal Medicine, School of Medicine, College of Medicine and Health Sciences Wollo University Dessie Ethiopia; ^3^ Department of Medical Laboratory Science, College of Medicine and Health Sciences Debre Berhan University Debre Berhan Ethiopia; ^4^ Department of Midwifery, School of Nursing and Midwifery College of Medicine and Health Sciences Wollo University Dessie Ethiopia

**Keywords:** associated factors, asymptomatic malaria, Ethiopia, malaria, prevalence

## Abstract

**Background and Aims:**

Malaria elimination programs have also encountered numerous challenges, such as widespread asymptomatic carriers in endemic areas, which should be taken into account in malaria‐control programs for effective transmission interruption. The purpose of this research was to determine the prevalence of symptomatic and asymptomatic malaria infections and associated factors, in pastoral communities.

**Methods:**

A community‐based cross‐sectional study was conducted among selected districts in the Waghemra Zone, Northeast Ethiopia, from September to December 2022. A structured questionnaire was employed to collect sociodemographic data and associated risk factors. *Plasmodium* species were detected using light microscopy and a rapid diagnostic test. Data entry and analysis were carried out using SPSS version 26 software. The association between dependent and independent variables was explored by using multivariable logistic regression analyses. A statistically significant association was declared at a *p*‐value of <0.05.

**Results:**

The overall prevalence of malaria was 21.2% (134/633), with the predominant *Plasmodium falciparum* infections accounting for 67.8% (87/134). Among asymptomatic participants, 7.5% (34/451) and 10.2% (46/451) were diagnosed by rapid diagnostic test and light microscopy, respectively. On the other hand, the prevalence of symptomatic malaria was 44.5% (81/182) and 48.4% (88/182) as diagnosed by rapid diagnostic test and light microscopy, respectively. The presence of stagnant water near the houses, the utilization of insecticide‐treated mosquito nets, the number of insecticide‐treated mosquito nets, and outdoor stays at night were all positively linked with the prevalence of malaria.

**Conclusions:**

The overall prevalence estimate for symptomatic and asymptomatic malaria was high. Malaria is still a public health problem in the study area. Malaria infection was associated with the presence of stagnant water near the houses, the utilization of insecticide‐treated mosquito nets, the number of insecticide‐treated mosquito nets, and outdoor stays at night. Improved access to all malaria interventions is needed to interrupt the transmission at the community level.

## 1 INTRODUCTION

Malaria is one of the most serious public health issues, as well as a potentially fatal parasitic disease caused by *Plasmodium* species.^1^ *Plasmodium* infection can produce symptoms ranging from asymptomatic to severe and fatal malaria.^2^, ^3^ Malaria control strategies implemented worldwide have resulted in significant reductions in disease morbidity and mortality, and Ethiopia is one of the countries that has had great success.^4^ Malaria incidence has decreased from 5.2 million in 2015 to less than 1 million in 2019. Malaria deaths fell from 3.6 to 0.3 per 100,000 people at risk.^5^, ^6^ However, Ethiopia still accounts for 1.7% of all malaria cases worldwide. In 2021, there were 2,783,816 cases and 8041 deaths.^7^

Malaria primarily affects the community during the planting and harvesting seasons, when agricultural labor is most needed. Healthcare institutions are overburdened with patients during epidemics, and many resources are diverted to deal with the situation. This places a major financial burden on poverty alleviation.^8^, ^9^ Ethiopia has launched a malaria elimination program with the goal of eliminating the disease by 2030. To achieve this objective, the government is now focusing on three key malaria prevention strategies: early diagnosis and rapid treatment; vector control methods such as indoor residual spraying (IRS) and insecticide‐treated bed nets (ITNs); and surveillance.^4^, ^10^

Malaria control and elimination are becoming increasingly difficult in many nations, including Ethiopia. The prevalence of asymptomatic carriers, the evolution of insecticide‐resistant vectors and drug‐resistant *Plasmodium* species, and a lack of long‐term and stable financing are among the challenges.^7^ Asymptomatic malaria infections are almost certainly a significant parasite reservoir, enabling parasite transmission to continue.^11^, ^12^ This is due to the fact that, asymptomatic carrier parasites are more infectious to the *Anopheles* mosquito and serve as a major source of gametocytes for local mosquito vectors, which helps to maintain malaria spread.^13^, ^14^ These factors hampered the government's attempts to control and eliminate malaria.

In high‐endemic areas, asymptomatic malaria infection is more prevalent.^15^ Active surveillance is frequently used to detect asymptomatic malaria infections. Furthermore, a small proportion of infected individuals is sufficient to restart malaria transmission in regions where malaria transmission is seasonal and low.^16^, ^17^ In addition to other preventive and control measures, the national malaria elimination program should target the identification of both symptomatic and asymptomatic malaria in communities and treat them with appropriate anti‐malarial medications.^18^, ^19^ This is especially true in the Waghemra Zone, where both symptomatic and asymptomatic malaria are prevalent.

Despite the fact that some research on asymptomatic or symptomatic both types of malaria are conducted in various locations of Ethiopia, they often focus on health institution‐based studies and pregnant women. As a result, understanding the present prevalence of symptomatic and asymptomatic malaria, as well as the risk factors linked with it, in communities is critical for scaling up intervention programs. In the study area, there has been no research on the prevalence of symptomatic and asymptomatic malaria. As a result, the purpose of this study was to determine the prevalence of symptomatic and asymptomatic malaria, as well as associated factors, among pastoral communities in the Waghemra Zone.

## 2 MATERIALS AND METHODS

### 2.1 Study design, period, and area

A community‐based cross‐sectional study was conducted from September to December 2022 among selected districts in the Waghemra Zone. Waghemra Zone is one of the 11 zones in Amhara region, Ethiopia. It is located at the following latitude and longitude coordinates: 12° 45′ 54″ N, 38° 50′ 35″ E. This zone is divided into eight districts with a total population of 536,129 people. Three districts in the Waghemra zone are highly malarious but differ in magnitude. The area's average yearly temperature and rainfall are 26°C and 786 mL, respectively. The climate and topography of the locations are ideal for the breeding of *Anopheles* mosquitoes. Furthermore, the Tekeze, Bargba, and Tela Rivers have several irrigation and construction dams that are used for mosquito reproduction. Therefore, malaria is one of the leading causes of morbidity and mortality in those districts. The main occupations in the districts are subsistence farming, livestock rearing, and fishing.

### 2.2 Sample size determination

The sample size was calculated using a single population proportion formula, the 95% confidence interval (CI) (Zα/2 = 1:96), and 5% margin of error (d) with a maximum proportion of 50%. Sample size = *Z*(*α*/2)^2^ *P* (1−*P*)/*d* ^2^, (1.96)^2^ *0.221 (1–0.221)/(0.05)^2^ = 384. Accounting for a 10% nonresponse rate and implementing design effect of 1.5, the final sample size was 633.

### 2.3 Sampling techniques and procedure

A stratified sampling method was used to select the representative sample size. The total households for each village were available at their nearest health post, which is stored as a family card folder, to determine the proportional sample size for each selected kebele. Based on this, the total sample size was proportionally allocated as 146, 104, 127, 81, 114, and 61 households to Tsitsika, Netsawork, Silazge, Meharit, Saka, and Debre‐brihan kebeles, respectively. Each household was selected using a systematic random sampling technique. Finally, one of the selected household members was chosen at random using a lottery method.

### 2.4 Inclusion and exclusion criteria

Individuals with and without clinical malaria symptoms who are permanent residents of the districts were included in the study. On the other hand, individuals who had taken antimalarial medications in the 4 weeks before the research period were excluded.

### 2.5 Data collection methods

#### 2.5.1 Sociodemographic and other factors

A structured questionnaire was used to collect information from each participant, including associated factors such as background characteristics, behavioral, environmental, and other factors relevant to malaria infection. Selected adult participants were interviewed directly; for children, parents were involved in the interview process. After the selection of the study participants in the households, body temperature and other clinical manifestations were examined by senior BSc nurses to identify symptomatic and asymptomatic individuals.

#### 2.5.2 Blood sample collection, transportation, and examination

In addition to sociodemographic and other variables, blood samples were taken aseptically from each subject via a finger prick with a disposable sterile lancet. Rapid diagnostic test (RDT) and blood films were used to screen the samples for malaria parasites.

##### RDT (CareStartTM malaria Pf/Pv [HRP2/pLDH]) Ag combo test

After the bag was opened, the capillary sample was wiped onto the sample pad of the test kit for instant use. After about two drops of buffer were placed in the buffer pad, the result was read after 20 min.^20^

##### Blood film examinations

For each participant, thin and thick blood films were produced on the same slide and labeled with a unique code for the microscopic study of *Plasmodium* spp. The thin blood smear was fixed after drying by delicately dripping methanol with a Pasteur pipette. The methanol‐fixed thin smears were permitted to dry completely in the air by placing the slides on a level surface (about 2 min). The blood smears were transported to nearby health facilities in a slide box for giemsa staining. At least 100 high‐power fields (100 objectives) were assessed before reporting a negative result.^21^

#### 2.5.3 Quality control

The questionnaire was pretested, and the data collectors received training. The expiration dates of the RDT kits were examined. The manufacturer's instructions were strictly followed for the RDTs. Positive and negative blood films were run to ensure the quality and integrity of the giemsa staining. Two experienced laboratory technologists individually examined the microscopic slides. Hundred microscopic fields of the thick smear were examined before concluding as negative. The discrepancy between the first and second readings was settled by a third senior laboratory technologist. Blood smear microscopy readers was blinded to the results of RDTs. Houses that were absent during the first visit were revisited again in the following day to ensure maximum involvement. All test methods and results were interpreted in accordance with standard operating procedures.

#### 2.5.4 Data analysis and interpretation

Following data collection, SPSS version 26 was used to enter and analyze the data. Descriptive statistics were used to assess the prevalence rate and determining factors. To assess an association of factors with malaria infection, bivariable and multivariable logistic regression models were utilized. In the bivariable analysis, variables having a *p*‐value of less than or equal to 0.25 were included in the multivariable logistic regression analysis. The 95% CI was used to determine the odds ratio (OR), and a *p*‐value of less than 0.05 was considered statistically significant. Finally, the study's findings were presented in text and tables as appropriate.

## 3 RESULTS

### 3.1 Sociodemographic characteristics of study participants

In this study a total of 633 asymptomatic and symptomatic patients, with a 100% response rate were included. In terms of age category, 43.4% (275/633) of participants were between the ages of 15 and 45. Of the overall study participants, 48.5% (307/633) were farmers, and 79.3% (502/633) had a family size of 4−6 people (Table 1).

**Table 1:** Sociodemographic characteristics of study participants in Waghemra Zone, Northeast Ethiopia, 2022.

Variables	Alternatives	Total number (%)
Sex	Male	341 (53.9)
	Female	292 (46.1)
Age	<5	85 (13.4)
	5−14	153 (24.2)
	15−45	275 (43.4)
	>45	120 (19.0)
Occupation	Government employee	31 (4.9)
	Unemployed	22 (3.5)
	Student	186 (29.4)
	Pastoralist	87 (13.7)
	Farmer	307 (48.5)
Educational status	Illiterate	346 (54.7)
	Primary	222 (35.1)
	Secondary and above	65 (10.3)
Family size	<3	61 (9.6)
	4−6	502 (79.3)
	>6	70 (11.1)
Kebeles name	Tsitsika	146 (23.1)
	Netsawork	104 (16.4)
	Silazge	127 (20.1)
	Meharit	81 (12.8)
	Saka	114 (18.0)
	Debre‐brihan	61 (9.6)

### 3.2 The overall prevalence of malaria

Malaria was found in 21.2% (134/633) of symptomatic and asymptomatic subjects in the Waghemra Zone (95% CI = 18.0−25.0). Of these, 56.0% (75/134) were males and 44.0% (59/134) were females. Similarly, the prevalence of malaria was greater in the age groups of 15−45 and 5−14 years, with prevalence rates of 35.1% (47/134) and 30.6% (41/134), respectively (Table 2).

**Table 2:** Sociodemographic variables and malaria infection among participants in Waghemira zone, Northeast Ethiopia, 2022.

Variables	Alternatives	Total tested	Malaria	
			Positive	Negative
		Number (%)	Number (%)	Number (%)
Sex	Male	341 (53.9)	75 (22.0)	266 (78.0)
	Female	292 (46.1)	59 (20.2)	233 (79.8)
Age	<5	85 (13.4)	21 (24.7)	64 (75.3)
	5−14	153 (24.2)	41 (26.8)	112 (73.2)
	15−45	275 (43.4)	47 (17.1)	228 (82.9)
	>45	120 (19.0)	25 (20.8)	95 (79.2)
Occupation	Government employee	31 (4.9)	7 (22.6)	24 (77.4)
	Unemployed	22 (3.5)	5 (22.7)	17 (77.3)
	Student	186 (29.4)	36 (19.4)	150 (80.6)
	Pastoralist	87 (13.7)	15 (17.2)	72 (82.8)
	Farmer	307 (48.5)	71 (23.1)	236 (76.9)
Educational status	Illiterate	346 (54.7)	86 (24.9)	260 (75.1)
	Primary	222 (35.1)	38 (17.1)	184 (82.9)
	Secondary and above	65 (10.3)	10 (15.4)	55 (84.6)
Family size	<3	61 (9.6)	14 (23.0)	47 (77.0)
	4−6	502 (79.3)	84 (16.7)	418 (83.3)
	>6	70 (11.1)	36 (51.4)	34 (48.6)
Kebeles name	Tsitsika	146 (23.1)	29 (19.9)	117 (80.1)
	Netsawork	104 (16.4)	20 (19.2)	84 (80.8)
	Silazge	127 (20.1)	20 (15.7)	107 (84.3)
	Meharit	81 (12.8)	12 (14.8)	69 (85.2)
	Saka	114 (18.0)	35 (30.7)	79 (69.3)
	Debre‐brihan	61 (9.6)	18 (29.5)	43 (70.5)
Overall total		633 (100.0)	134 (21.2)	499 (78.8)

### 3.3 Asymptomatic and symptomatic malaria

From a total of 633 study participants, 451 (71.2%) were asymptomatic and the remaining 182 (28.8%) were symptomatic. Overall, the prevalence of malaria diagnosed by RDT and microscopy was 18.2% (115/633) (95% CI: 14.6−22.4) and 21.2% (134/633) (95% CI: 18.0−25.0), respectively. Malaria prevalence among asymptomatic and symptomatic individuals was 10.2% (46/451) and 48.4% (88/182), respectively (Table 3).

**Table 3:** Malaria detection by rapid diagnostic test and microscopy in Waghemra zone, Northeast Ethiopia from September to November, 2022.

Participant type	Number (%)	Diagnostic test		
		RDT	Microscopy	Total
		Positive, *n* (%)	Positive, *n* (%)	Positive, *n* (%)
Asymptomatic	451 (71.2)	34 (7.5)	46 (10.2)	46 (10.2)
Symptomatic	182 (28.8)	81 (44.5)	88 (48.4)	88 (48.4)
Overall total	633 (100.0)	115 (18.2)	134 (21.2)	134 (21.2)

Abbreviation: RDT, rapid diagnostic test.

Among 115 asymptomatic and symptomatic malaria‐infected individuals detected using RDT in the survey, *Plasmodium falciparum*, *Plasmodium vivax*, and mixed accounted for 67.8%, 29.6%, and 2.6%, respectively. *P. falciparum*, *P. vivax*, and mixed parasites were found in 65.0%, 31.3%, and 3.7% of 134 microscopically confirmed asymptomatic and symptomatic malaria cases, respectively. All RDT‐positive cases were also positive using microscopy (Table 4).

**Table 4:** The relative proportion of *Plasmodium* species among confirmed cases by RDT and light microscopy in Waghemra zone, Northeast Ethiopia, September to November, 2022.

Malaria	RDT				Microscopy			
	PF (%)	PV (%)	Mix (%)	Total positive	PF (%)	PV (%)	Mix (%)	Total positive
Asymptomatic	22 (64.7)	11 (32.4)	1 (2.9)	34	29 (63.1)	15 (32.6)	2 (4.3)	46
Symptomatic	56 (69.1)	23 (28.4)	2 (2.5)	81	58 (65.9)	27 (30.7)	3 (3.4)	88
Overall total	78 (67.8)	34 (29.6)	3 (2.6)	115	87 (65.0)	42 (31.3)	5 (3.7)	134

Abbreviation: RDT, rapid diagnostic test.

### 3.4 Factors associated with malaria infection

Out of the screened participants, 55.5% (351/633) reported having slept under ITNs on a regular basis during the previous 2 weeks. IRS coverage and frequency were assessed during the survey, but none of the households were sprayed. In bivariable logistic regression, previous history of malaria infection, stagnant water near the house, ITN utilization, number of ITNs, outdoor stay at night, presence of a hole on the wall of the house, and knowledge of malaria transmission and prevention were chosen and entered into the forward stepwise multivariable logistic regression model.

In multivariable logistic regression analysis, the presence of stagnant water near the house, ITN utilization, number of ITNs, and outdoor stay at night were found to have a statistically significant association with malaria infection. People who were living near a mosquito breeding site at a distance of ~1 km were nearly seven times more at risk of getting malaria infection than their counterparts (adjusted OR [AOR]: 6.56; 95% CI: 4.085−10.539). Similarly, those respondents who did not use ITNs regularly were at about four times higher risk of developing malaria compared to regularly used (AOR: 4.389; 95% CI: 2.763−6.972). Likewise, the odds of contracting malaria among respondents who lived in households with an inadequate number of ITNs per family size were about three times higher than their counterparts (AOR: 3.23; 95% CI: 1.918−5.425). Moreover, individuals who stayed outside at night had an almost twofold greater risk of acquiring malaria compared to those who did not (AOR = 1.85; 95% CI: 1.013−3.361) (Table 5).

**Table 5:** Factors associated with malaria in Waghemra zone, Northeast Ethiopia, 2022.

Variables	Alternatives	Malaria status			COR (95% CI) *p*	AOR (95% CI) *p*
		Positive (%)	Negative (%)	Total (%)		
Sex	Male	75 (22.0)	266 (78.0)	341 (53.9)	1.113 (0.759−1.634) 0.583	
	Female	59 (20.2)	233 (79.8)	292 (46.1)	1	
Age	<5	21 (24.7)	64 (75.3)	85 (13.4)	1.592 (0.887−2.855) 0.319	
	5−14	41 (26.8)	112 (73.2)	153 (24.2)	0.896 (0.487−1.648) 0.525	
	15−45	47 (17.1)	228 (82.9)	275 (43.4)	0.896 (0.487−1.648) 0.725	
	>45	25 (20.8)	95 (79.2)	120 (19.0)	1	
Previous history of malaria	Yes	40 (28.2)	102 (71.8)	142 (22.4)	1.656 (1.078−2.544) 0.021^a^	0.657 (0.362−1.191) 0.166
	No	94 (19.1)	397 (80.9)	491 (77.6)		
Stagnant water near to the house	Yes	100 (39.8)	151 (60.2)	251 (39.7)	6.778 (4.394−10.456) <0.001^a^	6.561 (4.085−10.539) <0.001^b^
	No	34 (8.9)	348 (91.1)	382 (60.3)	1	
Distance from health center (h)	≤1	40 (18.6)	175 (81.4)	215 (34.0)	1	
	>1	94 (22.5)	324 (77.5)	418 (66.0)	1.269 (0.840−1.919) 0.258	
Proper utilization of ITNs	Yes	42 (12.0)	309 (88.0)	351 (55.5)	1	
	No	92 (32.6)	190 (67.4)	282 (44.5)	3.562 (2.371−5.353) <0.001^a^	4.389 (2.763−6.972) <0.001^b^
Number of ITNs	Adequate	24 (10.4)	207 (89.6)	231 (36.5)	1	
	Inadequate	110 (27.4)	292 (72.6)	402 (63.5)	3.249 (2.018−5.231) <0.001^a^	3.225 (1.918−5.425) <0.001^b^
Outdoor stay at night	Yes	31 (40.8)	45 (59.2)	76 (12.0)	3.036 (1.832−5.031) <0.001^a^	1.845 (1.013−3.361) 0.045^b^
	No	103 (18.5)	454 (81.5)	557 (88.0)	1	
Presence of hole on the wall of house	Yes	48 (30.4)	110 (69.6)	158 (25.0)	1.974 (1.308−2.979) <0.001^a^	0.915 (0.435−1.924) 0.814
	No	86 (18.1)	389 (81.9)	475 (75.0)	1	
Environmental management for malaria prevention	Yes	33 (24.4)	102 (75.6)	135 (21.3)	1	
	No	101 (20.3)	397 (79.7)	498 (78.7)	0.786 (0.502−1.232) 0.294	
Knowledge of malaria transmission and prevention	Good	67 (18.6)	293 (81.4)	360 (56.9)	1	
	Poor	67 (24.5)	206 (75.5)	273 (43.1)	1.422 (0.970−2.085) 0.071^a^	1.444 (0.917−2.272) 0.112

Abbreviations: AOR, adjusted odd ratio; COR, crude odd ratio; ITNs, insecticide‐treated mosquito nets.

^a^ Significant variable in bivariate analysis.

^b^ significant variables in multivariable analysis.

## 4 DISCUSSION

This study found that the overall prevalence of malaria in pastoral communities in the Waghemira Zone was 21.2% (95% CI = 18.0−25.0). It was comparable to a study conducted in the East Shewa Zone, Central Ethiopia (25%),^22^ Abaya district, Southern Ethiopia (21.9%),^23^ and Mizan‐Aman town, Southwest Ethiopia (21.1%).^24^ However, the prevalence of malaria infections found in this study was low as compared to a previous study conducted in Douala, Cameroon (30.1%),^25^ Mozambique (38.9%),^26^ Nigeria (41.6%),^27^ and Tanzania (36.3%).^28^ This disparity could be attributed to differences in malaria prevalence and burden across nations. Malaria prevalence in Ethiopia is low when compared to other endemic nations in sub‐Saharan Africa. Nigeria, Mozambique, and Tanzania, on the other hand, are among the top nations in terms of malaria cases and deaths.^10^

Lower malaria prevalence rates have been reported from various parts of Ethiopia, including Debre Elias district, Northwest Ethiopia (5.0%),^12^ Dembiya district, North‐western Ethiopia (3.5%),^29^ Dilla town, Southern Ethiopia (16%),^30^ several regions of Ethiopia (4.1%),^26^ and Benna Tsemay district, Southwest Ethiopia (6.1%).^31^ The discrepancy may be due to a difference in the study period, as the two previous studies conducted in the Debre Elias and Benna Tsemay districts of southwest Ethiopia were conducted during the minor malaria transmission period. In contrast to these studies, the current study was conducted during the major malaria transmission period, which may overestimate the prevalence. Moreover, in the last year, there has been an internal conflict in the study area, which has resulted in the deterioration of the health system, the interruption of anti‐malarial measures, and the migration of nonimmune people, which has kept malaria under control. Besides, in this study area, animal breeding was the most dominant occupation, and this may expose them to the bite of mosquitoes.

The present study showed that the prevalence of asymptomatic malaria in the Waghemra Zone was 10.2% (95% CI = 8.1−12.4). This finding was comparable with the study conducted in Jawi district, Northwest Ethiopia (11.2%),^15^ Gondar Zuria district, Northwest Ethiopia (12.0%),^32^ and in the lowlands of Ethiopia (9.3%).^33^ However, a higher prevalence of asymptomatic malaria was reported in Pawe, western Ethiopia (14.5%),^34^ Nigeria (77.6%),^35^ Douala, Cameroon (28.9%),^25^ and Bagamoyo district, Tanzania (57.5%).^28^ This difference could be attributed to geographical differences relative to the current study area, as the aforementioned area is extremely malarial and the population is constantly exposed to malaria, which could contribute to the development of immunity, resulting in asymptomatic malaria.^36^ Furthermore, the diagnostic method we used differed from those used in a Tanzanian study that used a molecular technique and in Douala, Cameroon, LED fluorescence microscopy was used for malaria detection, which is very sensitive; the asymptomatic infection has been missed by conventional malaria diagnostic tests due to low parasite density.^37^

The prevalence rate of asymptomatic malaria in this study was higher than the study done in Raya Kobo district, Northeast Ethiopia (7.0%),^11^ Dembia district, Northwest Ethiopia (6.7%),^38^ Debre Elias district, Northwest Ethiopia (4.2%),^12^ Sanja Town, Northwest Ethiopia (6.8%),^39^ Boset District, Central Ethiopia (3.05%),^40^ and Maygaba Town, Northwest Ethiopia (4.7%).^41^ The differences might be due to inadequate environmental and vector control interventions in affected areas. The national malaria prevention and control strategies recommend the application of the IRS at least once a year with 100% coverage and at least one ITN per two people in malaria high‐risk areas.^42^ Despite this fact, IRS was not applied before the study period, and early replacement of ITNs was not done. Households that had been using the ITNs for purposes other than their intended purpose were also observed. This could be due to poor monitoring of the communities after distributing the ITNs.

This study showed that the prevalence of symptomatic malaria in pastoral communities of Waghemra Zone was 48.4% (95% CI = 44.2−53.0). The prevalence of this study was lower than a study done in Ibadan, Nigeria (55.0%).^43^ This disparity could be attributed to differences in study design and setting; for example, the study in Ibadan, Nigeria, was done in health facilities, which may have overestimated the prevalence compared to a community‐based study. In contrary to this, the prevalence of symptomatic malaria in this study was higher than reported in Ziquala district, Northeast Ethiopia (24.6%),^44^ Lake Tana and its surrounding areas, Northwest Ethiopia 24.7%,^45^ the Mount Cameroon area (41.7%),^46^ the Pakro subdistrict of Ghana 32.9%,^47^ and Dzanga Sangha Region, Central African Republic (35.2%)^48^ and Malawi (19.0%).^49^ This variation might be attributed to the different data collection periods, settings, laboratory method, and laboratory personnel skills.

In general, the prevalence of malaria differed between symptomatic and asymptomatic individuals in this study. This disparity could be attributed to the fact that we used various sample sizes and that the diagnosis was performed using low‐sensitivity techniques, particularly for the diagnosis of asymptomatic malaria.^37^ Asymptomatic malaria patients have acquired partial immunity, which clears the parasite and results in a low parasite density compared to symptomatic patients. As a result, poor sensitivity techniques such as microscopy and RDT may miss detecting asymptomatic malaria.^37^, ^50^

This study revealed that the predominant *Plasmodium* species detected was *P. falciparum* (65.0%), followed by *P. vivax* (31.3%). This was in agreement with other previous studies done in the Argoba district, Northeast Ethiopia^51^ and the Abergele district, Northern Ethiopia.^52^ However, it disagreed with the national malaria parasite distribution pattern of Ethiopia^53^ which showed that *P. falciparum* and *P. vivax* accounted for 60% and 40% of the malaria cases in the country, respectively. This variation could be due to the fact that this study was limited to a small malaria‐endemic setting in the country, which could have caused the species prevalence to vary. Furthermore, because *P. falciparum* is a common species in the lowlands, there is the possibility of recrudescence.

In the present study, the presence of stagnant water, ITN utilization, number of ITNs and outdoor stay at night were found to have a statistically significant association with malaria infection. Furthermore, people who live near stagnant water are more likely to be exposed to the malaria parasite than those who do not live near stagnant water. There are numerous freshwater collections that formed during the rainy season, which were ideal for mosquito breeding. A similar finding was reported from a study done in Simada district, Northwest Ethiopia.^54^

This study also found that households that did not regularly use ITNs were 4.39 times more likely than those who used regularly to be infected with the malaria parasite. This result supported by similar previous studies conducted in South Gondar and Arbaminch Zuria districts, Ethiopia's.^55^, ^56^ ITN are an effective vector control method for preventing malaria transmission. When they use ITNs consistently, the risk of getting a mosquito bite might be decreased.^57^ As a result, in malaria‐endemic areas, local authorities must enforce regular ITN use.

Respondents who lived in households with an inadequate number of ITNs were 3.23 times more likely to develop malaria. Inadequate ITNs in households might enforce the community to utilize the ITNs infrequently. It is recommended that at least one ITN for every 2 persons in the household. Similarly, people who stayed outside at night were approximately five times more likely to be infected with malaria than those who did not. This finding was supported by a report from the Ziquala, Armachiho, and Dembia districts of the Amhara region of Ethiopia.^44^, ^58^, ^59^ This could be explained by the exophagic—exophilic biting behaviors of mosquitoes.^60^ This could also be attributed to the hot weather, as people prefer to sleep outside to get fresh air and cool off, and this could make it difficult to use ITNs while sleeping outside.

### 4.1 Limitation of the study

This study maintained its strength by using larger samples and sampling approaches to reduce selection bias and assure internal and external validity. Confounding factors were also eliminated with the help of binary logistic regression analysis. In this study, like any cross‐sectional study, it is difficult to establish a causal association. Furthermore, due to a shortage of resources, this study was unable to apply the molecular tool PCR for detecting malaria to support RDT and microscopic investigation.

## 5 CONCLUSION AND RECOMMENDATIONS

In this study, the overall prevalence estimate of symptomatic and asymptomatic malaria was high. Among *Plasmodium* species, *P. falciparum* was found to be the most prevalent in the study area. Due to internal conflict, for the last year of the study, none of the houses were sprayed by the IRS. Regarding associated factors, the presence of stagnant water near the houses, the utilization of ITNs, the number of ITNs, and outdoor stays at night were significantly associated with malaria infection.

The following recommendations are made based on the findings. Because ITN and IRS have potential protection gaps, their combination with environmental management interventions should be applied in the study area. Therefore, community awareness regarding ITN utilization and malaria prevention and control mechanism, and removal of potential mosquito breeding sites should be conducted regularly. Further prevalence studies should be conducted using highly sensitive and specific molecular methods. Both asymptomatic and symptomatic malaria should be considered during community malaria screening in the implementation of the prevention and control program so that the efficacy of control strategies can be assessed using reliable metrics.

## AUTHOR CONTRIBUTIONS

**Habtu Debash**: Conceptualization; data curation; formal analysis; funding acquisition; investigation; methodology; project administration; resources; software; supervision; validation; visualization; writing—original draft; writing—review and editing. **Gebru Tesfaw**: Conceptualization; data curation; formal analysis; funding acquisition; investigation; methodology; project administration; resources; software; supervision; validation; visualization; writing—review and editing. **Hussen Ebrahim**: Conceptualization; data curation; formal analysis; funding acquisition; investigation; methodology; project administration; resources; software; supervision; validation; visualization. **Agumas Shibabaw**: Conceptualization; data curation; formal analysis; funding acquisition; investigation; methodology; resources; software; supervision; validation; visualization; writing—review and editing. **Yimer Melese**: Conceptualization; data curation; formal analysis; funding acquisition; investigation; methodology; resources; software; supervision; validation; visualization; writing—review and editing. **Mihret Tilahun**: Conceptualization; data curation; formal analysis; funding acquisition; investigation; methodology; project administration; resources; software; supervision; validation; visualization. **Ermiyas Alemayehu**: Conceptualization; data curation; formal analysis; funding acquisition; investigation; methodology; resources; software; supervision; validation; visualization. **Ousman Mohammed**: Conceptualization; data curation; formal analysis; funding acquisition; investigation; methodology; resources; software; supervision; validation; visualization. **Melkam Tesfaye**: Conceptualization; data curation; formal analysis; funding acquisition; investigation; methodology; resources; software; supervision; validation; visualization. **Mengistu Abate**: Conceptualization; data curation; formal analysis; funding acquisition; investigation; methodology; project administration; resources; software; supervision; validation; visualization.

## CONFLICT OF INTEREST STATEMENT

The authors declare no conflict of interest.

## ETHICS STATEMENT

Ethical clearance was obtained from the ethical review committee of College of Medicine and Health Sciences, Wollo University on the date 16/8/2022 with a protocol number of CMHS/201/2022. Permission was obtained from Waghemra Zone Health Office and each district health office where the study was conducted. This study was conducted in accordance with the Declaration of Helsinki. After briefly describing the significance of the study, the participants or children's parents or guardians signed informed written consent. Confidentiality of the data was maintained. Finally, children who were infected with the *Plasmodium* parasite received antimalarial treatment according to the national malaria treatment guidelines.

## TRANSPARENCY STATEMENT

The lead author Habtu Debash affirms that this manuscript is an honest, accurate, and transparent account of the study being reported; that no important aspects of the study have been omitted; and that any discrepancies from the study as planned (and, if relevant, registered) have been explained.

## Data Availability

All relevant data are included in the published article.

## References

[1] Zareen S , Rehman HU , Gul N , et al. Malaria is still a life threatening disease review. J Entomol Zool Studies. 2016;4(5):105‐112.

[2] Laishram DD , Sutton PL , Nanda N , et al. The complexities of malaria disease manifestations with a focus on asymptomatic malaria. Malar J. 2012;11(1):29. 22289302 10.1186/1475-2875-11-29 PMC3342920

[3] Chen I , Clarke SE , Gosling R , et al. “Asymptomatic” malaria: a chronic and debilitating infection that should be treated. PLoS Med. 2016;13(1):e1001942. 26783752 10.1371/journal.pmed.1001942 PMC4718522

[4] Bugssa G , Tedla K . Feasibility of malaria elimination in Ethiopia. Ethiop J Health Sci. 2020;30(4):607‐614. 33897221 10.4314/ejhs.v30i4.16 PMC8054453

[5] Federal Ministry of Health . National malaria strategic plan 2017–2020. Addis Ababa, Ethiopia: Ministry of Health. 2017. https://www.medbox.org/national-malaria-guidelines-ethiopia/download.pdf

[6] Aregawi M , Lynch M , Bekele W , et al. Time series analysis of trends in malaria cases and deaths at hospitals and the effect of antimalarial interventions, 2001–2011. Ethiopia PLoS ONE. 2014;9:e106359. 25406083 10.1371/journal.pone.0106359 PMC4236017

[7] WHO . World Malaria Report. World Health Organization; 2022.

[8] Taffese HS , Hemming‐Schroeder E , Koepfli C , et al. Malaria epidemiology and interventions in Ethiopia from 2001 to 2016. Infect Dis Poverty. 2018;7(6):103. 30392470 10.1186/s40249-018-0487-3 PMC6217769

[9] Jima D , Getachew A , Bilak H , et al. Malaria indicator survey 2007, Ethiopia: coverage and use of major malaria prevention and control interventions. Malar J. 2010;9:58. 20178654 10.1186/1475-2875-9-58 PMC2841196

[10] World Health Organization . Global Technical Strategy for Malaria 2016‐2030. World Health Organization; 2015.

[11] Melese Y , Alemu M , Yimer M , Tegegne B , Tadele T . Asymptomatic malaria in households and neighbors of laboratory confirmed cases in Raya Kobo District, Northeast Ethiopia. Ethiop J Health Sci. 2022;32(3):623‐630. 35813680 10.4314/ejhs.v32i3.19 PMC9214748

[12] Abebaw A , Aschale Y , Kebede T , Hailu A . The prevalence of symptomatic and asymptomatic malaria and its associated factors in Debre Elias district communities, Northwest Ethiopia. Malar J. 2022;21(1):167. 35659661 10.1186/s12936-022-04194-7 PMC9166605

[13] Alves FP , Gil LHS , Marrelli MT , Ribolla PEM , Camargo EP , Pereira Da Silva LH . Asymptomatic carriers of *Plasmodium* spp. as infection source for malaria vector mosquitoes in the Brazilian Amazon. J Med Entomol. 2005;42(5):777‐779. 16363160 10.1093/jmedent/42.5.777

[14] Keven JB , Katusele M , Vinit R , et al. Nonrandom selection and multiple blood feeding of human hosts by *Anopheles* vectors: implications for malaria transmission in Papua New Guinea. Am J Trop Med Hyg. 2021;105(6):1747‐1758. 34583342 10.4269/ajtmh.21-0210 PMC8641310

[15] Tilahun A , Yimer M , Gelaye W , Tegegne B . Prevalence of asymptomatic *Plasmodium* species infection and associated factors among pregnant women attending antenatal care at Fendeka town health facilities, Jawi District, North west Ethiopia: a cross‐sectional study. PLoS One. 2020;15(4):e0231477. 32315341 10.1371/journal.pone.0231477 PMC7173768

[16] Cheaveau J , Mogollon DC , Mohon MAN , Golassa L , Yewhalaw D , Pillai DR . Asymptomatic malaria in the clinical and public health context. Expert Rev Anti Infect Ther. 2019;17(12):997‐1010. 31718324 10.1080/14787210.2019.1693259

[17] Kendie FA , Hailegebriel W/kiros T , Nibret Semegn E , Ferede MW . Prevalence of malaria among adults in Ethiopia: a systematic review and meta‐analysis. J Trop Med. 2021;2021:1‐9. 10.1155/2021/8863002 PMC7952180 33747096

[18] Tegegne Y , Asmelash D , Ambachew S , Eshetie S , Addisu A , Jejaw Zeleke A . The prevalence of malaria among pregnant women in Ethiopia: a systematic review and meta‐analysis. J Parasitol Res. 2019;2019:1‐9. 10.1155/2019/8396091 PMC6521389 31186950

[19] Tamiru A , Tolossa T , Regasa B , Mosisa G . Prevalence of asymptomatic malaria and associated factors in Ethiopia: systematic review and meta‐analysis. SAGE Open Med. 2022;10:205031212210880. 10.1177/20503121221088085 PMC9006361 35433001

[20] Woyessa A , Deressa W , Ali A , Lindtjørn B . Evaluation of CareStart™ malaria Pf/Pv combo test for *Plasmodium falciparum* and *Plasmodium vivax* malaria diagnosis in Butajira area, south‐central Ethiopia. Malar J. 2013;12(1):218. 23805822 10.1186/1475-2875-12-218 PMC3700775

[21] World Health Organization . Malaria Microscopy Quality Assurance Manual‐version 2. World Health Organization; 2016.

[22] Tadesse F , Fogarty AW , Deressa W . Prevalence and associated risk factors of malaria among adults in East Shewa Zone of Oromia Regional State, Ethiopia: a cross‐sectional study. BMC Public Health, 2018. 18(1): 1‐8. 10.1186/s12889-017-4577-0 PMC5513396 28716009

[23] Girma Worku O , Gondol BN . Prevalence of malaria and associated factors among households in Guanga, Abaya District, Oromia Regional State, Southern Ethiopia: a community based cross‐sectional study. Health Sys Policy Res. 2021;8(5):93.

[24] Duguma T , Nuri A , Melaku Y . Prevalence of malaria and associated risk factors among the community of Mizan‐Aman town and its catchment area in Southwest Ethiopia. J Parasitol Res. 2022;2022:1‐8. 10.1155/2022/3503317 PMC9019455 35464173

[25] Mbohou CN , Foko LPK , Nyabeyeu HN , et al. Malaria screening at the workplace in Cameroon. PLoS One. 2019;14(12):e0225219. 31821328 10.1371/journal.pone.0225219 PMC6903749

[26] Ejigu BA . Geostatistical analysis and mapping of malaria risk in children of Mozambique. PLoS One. 2020;15(11):e0241680. 33166322 10.1371/journal.pone.0241680 PMC7652261

[27] Fana SA , Bunza MDA , Anka SA , Imam AU , Nataala SU . Prevalence and risk factors associated with malaria infection among pregnant women in a semi‐urban community of north‐western Nigeria. Infect Dis Poverty. 2015;4(1):24. 26269742 10.1186/s40249-015-0054-0 PMC4534061

[28] Sumari D , Mwingira F , Selemani M , Mugasa J , Mugittu K , Gwakisa P . Malaria prevalence in asymptomatic and symptomatic children in Kiwangwa, Bagamoyo district, Tanzania. Malar J. 2017;16(1):222. 28545457 10.1186/s12936-017-1870-4 PMC5445421

[29] Tarekegn M , Tekie H , Dugassa S , Wolde‐Hawariat Y . Malaria prevalence and associated risk factors in Dembiya district, North‐western Ethiopia. Malar J. 2021;20:372. 34535130 10.1186/s12936-021-03906-9 PMC8447688

[30] Molla E , Ayele B . Prevalence of malaria and associated factors in Dilla town and the surrounding rural areas, Gedeo Zone, Southern Ethiopia. J Bacteriol Parasitol. 2015;6(5):1.

[31] Debo GW , Kassa DH . Prevalence of malaria and associated factors in Benna Tsemay district of pastoralist community, Southern Ethiopia. Trop Dis Travel Med Vaccines. 2016;2(1):16. 28883960 10.1186/s40794-016-0033-x PMC5530935

[32] Minwuyelet A , Eshetu T , Milikit D , Aschale Y . Prevalence and risk factors of asymptomatic *Plasmodium* infection in Gondar Zuria District, Northwest Ethiopia. Infect Drug Resist. 2020;13:3969‐3975. 33177847 10.2147/IDR.S278932 PMC7649210

[33] Goshu EM , Zerefa MD , Tola HH . Occurrence of asymptomatic malaria infection and living conditions in the lowlands of Ethiopia: a community‐based cross‐sectional study. Infect Dis Poverty. 2022;11(5):94. 36064653 10.1186/s40249-022-01018-3 PMC9444277

[34] Beyene H , Telele N , Mekuria A . Asymptomatic malaria and associated factors in Pawe, Northern Ethiopia. Int J Infec Tropical Dis. 2015;2(2):60‐69.

[35] Chukwudi O , Igwe N , Joannes UU , et al. Prevalence and parasite density of asymptomatic malaria parasitemia among unbooked paturients at Abakaliki, Nigeria. J Basic Clin Repr Sci. 2014;3(1):44‐48.

[36] Lindblade KA , Steinhardt L , Samuels A , Kachur SP , Slutsker L . The silent threat: asymptomatic parasitemia and malaria transmission. Expert Rev Anti Infect Ther. 2013;11(6):623‐639. 23750733 10.1586/eri.13.45

[37] Bottius E , Guanzirolli A , Trape JF , Rogier C , Konate L , Druilhe P . Malaria: even more chronic in nature than previously thought; evidence for subpatent parasitaemia detectable by the polymerase chain reaction. Trans R Soc Trop Med Hyg. 1996;90(1):15‐19. 8730301 10.1016/s0035-9203(96)90463-0

[38] Fekadu M , Yenit MK , Lakew AM . The prevalence of asymptomatic malaria parasitemia and associated factors among adults in Dembia district, northwest Ethiopia, 2017. Arch Public Health. 2018;76(1):74. 30598821 10.1186/s13690-018-0323-z PMC6300894

[39] Worku L , Damtie D , Endris M , Getie S , Aemero M . Asymptomatic malaria and associated risk factors among school children in Sanja town, Northwest Ethiopia. Int Sch Res Notices. 2014;2014:1‐6. 10.1155/2014/303269 PMC4897416 27355032

[40] Balcha F , Menna T , Lombamo F . Prevalence of asymptomatic malaria and associated factors among pregnant women at Boset District in East Shoa Zone, Oromia Region, Ethiopia: a cross‐sectional study. Malar J. 2023;22(1):28. 36698185 10.1186/s12936-023-04460-2 PMC9878930

[41] Yhdego TG , Gardew AD , Yifat FT . Malaria prevalence, knowledge and associated factors among household heads in Maygaba town, Ethiopia. PLOS Global Public Health. 2022;2(3):e0000071. 36962259 10.1371/journal.pgph.0000071 PMC10022035

[42] Gari T , Lindtjørn B . Reshaping the vector control strategy for malaria elimination in Ethiopia in the context of current evidence and new tools: opportunities and challenges. Malar J. 2018;17(1):454. 30518395 10.1186/s12936-018-2607-8 PMC6282332

[43] Awosolu OB , Yahaya ZS , Farah Haziqah MT , Simon‐Oke IA , Fakunle C . A cross‐sectional study of the prevalence, density, and risk factors associated with malaria transmission in urban communities of Ibadan, Southwestern Nigeria. Heliyon. 2021;7(1):e05975. 33521357 10.1016/j.heliyon.2021.e05975 PMC7820925

[44] Debash H , Bisetegn H , Ebrahim H , et al. Prevalence and associated risk factors of malaria among febrile under‐five children visiting health facilities in Ziquala district, Northeast Ethiopia: a multicenter cross‐sectional study. PLoS One. 2022;17(10):e0276899. 36301956 10.1371/journal.pone.0276899 PMC9612493

[45] Adugna F , Wale M , Nibret E . Prevalence of malaria and its risk factors in Lake Tana and surrounding areas, northwest Ethiopia. Malar J. 2022;21(1):313. 36333723 10.1186/s12936-022-04310-7 PMC9636828

[46] Teh RN , Sumbele IUN , Meduke DN , Ojong ST , Kimbi HK . Malaria parasitaemia, anaemia and malnutrition in children less than 15 years residing in different altitudes along the slope of Mount Cameroon: prevalence, intensity and risk factors. Malar J. 2018;17(1):336. 30249261 10.1186/s12936-018-2492-1 PMC6154899

[47] Ndong IC , Okyere D , Enos JY , et al. Prevalence of asymptomatic malaria parasitaemia following mass testing and treatment in Pakro sub‐district of Ghana. BMC Public Health. 2019;19:1622. 31795981 10.1186/s12889-019-7986-4 PMC6889629

[48] Korzeniewski K , Bylicka‐Szczepanowska E , Lass A . Prevalence of asymptomatic malaria infections in seemingly healthy children, the rural Dzanga Sangha region, Central African Republic. Int J Environ Res Public Health. 2021;18(2):814. 33477889 10.3390/ijerph18020814 PMC7833374

[49] Topazian HM , Gumbo A , Puerto‐Meredith S , et al. Asymptomatic *Plasmodium* falciparum malaria prevalence among adolescents and adults in Malawi, 2015–2016. Sci Rep. 2020;10(1):18740. 33127922 10.1038/s41598-020-75261-9 PMC7603306

[50] Girma S , Cheaveau J , Mohon AN , et al. Prevalence and epidemiological characteristics of asymptomatic malaria based on ultrasensitive diagnostics: a cross‐sectional study. Clin Infect Dis. 2019;69(6):1003‐1010. 30475992 10.1093/cid/ciy1005

[51] Tesema GA , Lakew AM , Akalu TY. Malaria outbreak investigation in Argoba District, South Wello Zone, Northeast Ethiopia, 2016: a case control study. 2019.

[52] Tesfay K , Assefa B , Addisu A . Malaria outbreak investigation in Tanquae Abergelle district, Tigray region of Ethiopia: a case–control study. BMC Res Notes. 2019;12(1):645. 31585549 10.1186/s13104-019-4680-7 PMC6778373

[53] Assefa A. The third Ethiopian malaria indicator survey 2015 (EMIS‐2015). In 28th Annual conference, 2016. 2016.

[54] Workineh B , Mekonnen FA , Sisay M , Gonete KA . Malaria outbreak investigation and contracting factors in Simada District, Northwest Ethiopia: a case–control study. BMC Res Notes. 2019;12(1):280. 31101068 10.1186/s13104-019-4315-z PMC6525450

[55] Workineh L , Lakew M , Dires S , et al. Prevalence of malaria and associated factors among children attending health institutions at South Gondar Zone, Northwest Ethiopia: a cross‐sectional study. Glob Pediatr Health. 2021;8:2333794X2110591. 10.1177/2333794X211059107 PMC8724997 34993279

[56] Abossie A , Yohanes T , Nedu A , Tafesse W , Damitie M . Prevalence of malaria and associated risk factors among febrile children under five years: a cross‐sectional study in Arba Minch Zuria District, South Ethiopia. Infect Drug Resist. 2020;13:363‐372. 32104008 10.2147/IDR.S223873 PMC7012238

[57] Karunamoorthi K . Vector control: a cornerstone in the malaria elimination campaign. Clin Microbiol Infect. 2011;17(11):1608‐1616. 21996100 10.1111/j.1469-0691.2011.03664.x

[58] Aschale Y , Mengist A , Bitew A , Kassie B , Talie A . Prevalence of malaria and associated risk factors among asymptomatic migrant laborers in West Armachiho District, Northwest Ethiopia. Res Rep Tropical Med. 2018;9:95‐101. 10.2147/RRTM.S165260 PMC6047623 30050360

[59] Agegnehu F , Shimeka A , Berihun F , Tamir M . Determinants of malaria infection in Dembia district, Northwest Ethiopia: a case‐control study. BMC Public Health. 2018;18(1):480. 29642899 10.1186/s12889-018-5370-4 PMC5896134

[60] Bilal MEES . Mapping of *Anopheles* mosquitoes (Diptera, Culicidae) in West Kassala, Wad Al Hileio and Khashm El Girba Localities, Kassala State, Sudan (2014−2015). University of Gezira. 2018.

